# Diagnostic Errors in Obstetric Morbidity and Mortality: Methods for and Challenges in Seeking Diagnostic Excellence

**DOI:** 10.3390/jcm13144245

**Published:** 2024-07-20

**Authors:** Nicole M. Krenitsky, India Perez-Urbano, Dena Goffman

**Affiliations:** Department of Obstetrics and Gynecology, Vagelos College of Physicians, Columbia University, New York, NY 10023, USA; nk2804@cumc.columbia.edu (N.M.K.); ip2443@cumc.columbia.edu (I.P.-U.)

**Keywords:** diagnostic errors, obstetrics, maternal morbidity, maternal mortality

## Abstract

Pregnancy-related morbidity and mortality remain high across the United States, with the majority of deaths being deemed preventable. Misdiagnosis and delay in diagnosis are thought to be significant contributors to preventable harm. These diagnostic errors in obstetrics are understudied. Presented here are five selected research methods to ascertain the rates of and harm associated with diagnostic errors and the pros and cons of each. These methodologies include clinicopathologic autopsy studies, retrospective chart reviews based on clinical criteria, obstetric simulations, pregnancy-related harm case reviews, and malpractice and administrative claim database research. We then present a framework for a future study of diagnostic errors and the pursuit of diagnostic excellence in obstetrics: (1) defining and capturing diagnostic errors, (2) targeting bias in diagnostic processes, (3) implementing and monitoring safety bundles, (4) leveraging electronic health record triggers for case reviews, (5) improving diagnostic skills via simulation training, and (6) publishing error rates and reduction strategies. Evaluation of the effectiveness of this framework to ascertain diagnostic error rates, as well as its impact on patient outcomes, is required.

## 1. Introduction

Pregnancy-related morbidity and mortality remain high across the United States (US), with stark racial disparities still persisting. Nationwide, in 2022, there were 817 deaths during pregnancy or during the 42 days after birth, yielding an overall mortality rate of 22.3 per 100,000 live births. This represents a decrease from 32.9 in 2021 and 23.8 in 2020 [1]. Regarding disparities, the mortality rate for Black, non-Hispanic birthing people was 49.5 per 100,000, 2.6 times that of White birthing people. In addition, for each obstetric death, an estimated 20 to 30 others experience morbidity [2,3]. Strikingly, the majority of deaths related to pregnancy are preventable. A 2020 Centers for Disease Control review of pregnancy-related deaths referred to obstetric mortality review committees across 38 states estimates preventability in 84% of the deaths reviewed [4].

Why are more and more pregnant people experiencing preventable death or serious harm in the childbirth process? In exploring factors leading to deaths during or after pregnancy, provider-level factors such as misdiagnosis and delay in diagnosis comprise a significant cause leading to mortality [5]. Given that the highest proportion of preventability for mortality lies in provider-level factors, a closer examination of the causes in this category is warranted. Perhaps obstetrics as a field has yet to focus on a key provider area to reduce harm: diagnostic errors.

Diagnostic errors in medicine are the failure to establish an accurate and timely explanation of a patient’s health problem or to communicate that explanation to the patient [6]. When dangerous obstetric conditions are under- or misdiagnosed, these errors represent a significant patient safety threat; it is impossible provide the correct treatment without the correct diagnosis. Research in general medicine estimates the incidence of diagnostic errors is 10 to 15%, with studies of hospital autopsies reporting major error rates of 8 to 24% [7,8,9]. This translates to over 12 million Americans estimated to be affected by diagnostic errors each year [10]. Among malpractice claims, diagnostic errors are the most common, most costly, and most dangerous medical mistakes [11]. With regard to the impact of diagnostic errors on patients, the first nationwide estimate of morbidity and mortality due to diagnostic errors was published in 2024, estimating 795,000 annual serious harms or deaths related to diagnostic errors [12]. In addition, diagnostic errors are costly, estimated to total more than USD 100 billion per year [13].

While the traditional notion of medical diagnosis conjures an internal process in a single doctor’s mind, today’s way of arriving at a diagnosis is a multistep, interdisciplinary process and collaboration between providers, patients, and the health environment and system. Medical diagnosis is a complex, inexact science with an inherent and variable measure of uncertainty. Thus, diagnostic errors often refer not to a provider’s lack of medical knowledge or error in judgment, but rather to failures and opportunities in health systems [14]. For example, a failure to diagnose postpartum depression may lie with a provider’s failure to recognize symptoms of depression evolving after birth, but it may also be due to a lack of systematic screening at the postpartum visit or poor patient and family education regarding postpartum depression warning signs. To take another example, delay in diagnosing postpartum hemorrhage is a leading cause of maternal morbidity and mortality. Delayed recognition of postpartum hemorrhage may occur due to a failure to investigate a patient’s report of early symptoms of hypovolemia, underappreciation of tachycardia given the physiologic rise in heart rate during pregnancy, the absence of quantification of blood loss, or a delay in blood product administration [15].

The various causes leading to missed or delayed obstetric diagnoses speak to the interdisciplinary and multifactorial nature of diagnostic errors. Frameworks such as the Safer Dx model highlight the system’s approach to diagnostic errors. The Safer Dx model utilizes the Donabedian structure-process-outcome model in which the structure is the complex adaptive sociotechnical system in which the diagnosis takes place [16]. It defines the sociotechnical dimensions of diagnostic errors, including team members, clinical context, workflow and communications, technology, organizational features, and the regulatory environment. It also clearly defines the components of the diagnostic process such as the patient–provider encounter, the performance and interpretation of diagnostic tests, follow up of diagnostic information, referrals, and patient-related factors. These factors lead to the intermediate outcome of safe diagnoses and the ultimate goal of improved patient outcomes.

Specific to obstetrics, prior studies suggest that provider-level factors contribute to a large proportion of harm during and after pregnancy [5]. Thus, a focus on the contribution of diagnostic errors in obstetrics has the potential to significantly reduce morbidity and mortality. Following the structure, process, and outcomes of diagnostic errors yields many opportunities for investigation; however, research on diagnostic errors in our field of obstetrics is extremely limited. To date, there are no nationwide estimates of diagnostic errors and harms in the field of obstetrics, and existing smaller studies are limited and largely international.

This paper seeks to emphasize this dearth of investigation into obstetric diagnostic errors, to highlight five selected research methods that can be utilized to determine obstetric diagnostic error and harm rates, and to propose six steps toward pursuing diagnostic excellence in obstetrics.

## 2. Methods: Selected Approaches to Identifying Diagnostic Errors

Across medical specialties, researchers have taken various approaches to estimate the rate and impact of diagnostic errors. In a review of the literature on diagnostic error rates, specifically in obstetrics, few articles exist worldwide, and even fewer specific to the United States. Five research methods proven in other specialties and emerging in obstetrics are detailed here, including their benefits and limitations. These include clinicopathologic autopsy studies, retrospective chart reviews, obstetric simulations and standardized patients, incident reporting and pregnancy mortality reviews, and malpractice and administrative claims studies. Examples of each method for obtaining diagnostic error rates and harm burdens are detailed below, and the pros and cons of each are summarized in Table 1. International studies are utilized when no domestic studies representative of a research method can be found. While the health care systems of the studied countries differ from the system of the United States, the methods of identifying diagnostic errors are applicable across borders.

(1) Clinicopathologic autopsy research

First, retrospective clinicopathologic studies using autopsy data can provide information on diagnostic errors for deaths [17,18,19]. In a systematic review of general autopsy series, the median error rate was 23.5% for major errors, suggesting that autopsies can reveal important unsuspected diagnoses [9]. This approach can capitalize on objective pathologic evidence to corroborate or dispel the working cause of death and therefore provide concrete information on diagnostic errors.

Published research on obstetric diagnostic errors from clinicopathologic studies is scarce. The following is an example of this method: a retrospective study of clinicopathologic discrepancies in obstetric mortality in Mozambique studied 91 obstetric-related deaths, and complete diagnostic autopsies were used as the gold standard to determine the cause of death. These were compared to the clinical diagnosis and discrepancies were classified as major and minor diagnostic errors. False-negative diagnoses were discrepancies for which the autopsy diagnosis was in the assessed diagnostic category, but the clinical diagnosis was in another diagnostic category. False-positive diagnoses were classified as discrepancies for which the clinical diagnosis was in the diagnostic category but not the autopsy diagnosis. The authors found that 38% had a clinicopathologic discrepancy. By category, the sensitivity for eclampsia was 100%, but the positive predictive value was only 33%. The sensitivity for peripartum infections was 17%, and the positive predictive value 50%. For obstetric hemorrhage, the sensitivity was 62%, with a positive predictive value of 95% [20]. Though this study was conducted in Mozambique, the US also utilizes medical examiners to conduct autopsies, the pathologic diagnoses of which can be compared to the clinical record death diagnoses.

The use of autopsy data to identify obstetric diagnostic errors is limited by the flaws inherent in the autopsy process and by the fact that only cases referred to and accepted by a medical examiner will be included. In addition to a robust referral system, the feasibility in this research method to detect diagnostic error relies on the capacity of medical examiners to take on obstetric cases. There may be bias in which cases are referred, and there is evidence of cognitive bias in forensic pathology determinations themselves [21]. Furthermore, this method provides diagnostic error rates for pregnancy-related mortality but does not include morbidity.

(2) Retrospective chart review of clinical criteria

Retrospective chart reviews provide an additional approach to discerning diagnostic errors. Clinical criteria such as concerning vital signs, abnormal laboratory values, and positive screening evaluations can be queried, and charts can be reviewed for an associated, or lack of associated, diagnosis and treatment plan [22]. One type of review involves screening patient charts for clinical evidence of morbidity that does not have an associated documented diagnosis. For example, a retrospective review of 5517 vaginal deliveries at a single hospital in France screened for a ten-point fall in hematocrit from predelivery, corresponding to a one-liter blood loss, in charts that did not have a diagnosis of postpartum hemorrhage. These patients were compared to those who were diagnosed with hemorrhage. Among screened patients, 90, or 1.63%, met the criteria for a ten-point hematocrit drop, suggesting the majority of hemorrhage leading to significant anemia was recognized. Missed diagnosis was related to the use of visual or estimated blood loss instead of quantitative blood loss [23].

The process of chart review for clinical evidence of an obstetric diagnosis creates an objective framework to discern diagnostic errors that is broadly applicable across health systems. Taking postpartum hemorrhage as an example, in addition to laboratory evidence of anemia, chart reviews can be utilized for quantitative blood loss. They can also be applied to other measures such as sepsis and hypertension criteria. One benefit of this process is that once implemented at an institution or across a health system, it is easily replicable. Moving from the idea of identification of diagnostic errors to prevention, chart reviews for missed diagnoses in the electronic medical record hold great potential to be converted from retrospective review processes to real-time clinical decision support and, even further, to prospective predictive modeling. This future direction, however, requires health informatics resources and thus may not be feasible for institutions without significant informatics support.

(3) Obstetric simulation and standardized patients

Another approach is to use simulations to study the misdiagnosis of obstetric conditions, as has been studied in other specialties [24,25]. For example, a cross-sectional study in birthing facilities in the Philippines crafted an identical simulated case for 103 obstetrics providers for cephalopelvic disproportion, postpartum hemorrhage, and pre-eclampsia. The overall rate of misdiagnosis was 29.8%. The most common scenarios included, cephalopelvic disproportion (in 25% of cases), postpartum hemorrhage (in 33% of cases), and pre-eclampsia (in 31% of cases) [26].

Simulation-based approaches to diagnostic errors have several benefits. They have the immediate advantage of allowing providers to receive real-time feedback on their diagnostic process. They can identify which obstetric conditions have the highest rates of diagnostic errors among providers undergoing a simulation to prioritize ongoing education efforts and performance improvement strategies. Knowledge gaps and systems issues brought to the surface via a simulation can generate diagnostic tools such as clinical algorithms, checklists, and electronic health record clinical decision support. Through these approaches, lessons learned from simulations can be broadly disseminated to multidisciplinary teams, even if not present for the simulation, and built into provider workflows. Obstetric simulations are already required, for example, by the Joint Commission; this research method may be achievable as it builds on an already existing process [27].

Simulations for diagnostic errors do not typically yield information on real patient cases and rates of diagnostic errors. However, a unique aspect of this study was linkage to real patient data at the providers’ health facilities. Medical charts of patients with obstetric complications at each participating provider’s facility were reviewed for diagnostic errors, and patient interviews were conducted for information on health outcomes and costs. The authors found an association between provider misdiagnosis in simulations and the presence of patient complications (OR 2.97, 95% CI 1.41, 3.32), worse outcomes, delays in referrals, and increased out-of-pocket patient costs. This novel method of linking simulation data to health system data and qualitative patient interviews may offer more robust information on which to build quality, patient safety, and performance improvement initiatives.

(4) Pregnancy-related or -associated morbidity and mortality case reviews

Pregnancy-related or -associated cases of severe morbidity and mortality are typically reviewed at the institutional level by severe obstetric morbidity and mortality reviews and root cause analyses, as well as at city or state levels by maternal mortality review committees (MMRCs). Institutional-level severe obstetric morbidity reviews and root cause analyses offer thorough individualized case reviews that can identify diagnostic errors and offer potential solutions to prevent future instances. The goal of these reviews is to seek and analyze comprehensive data from the case, determine whether the harm was associated with pregnancy, and develop recommendations to prevent similar harm in the future. MMRCs also provide comprehensive reviews but at the state or local levels. These multidisciplinary groups review death records identified from vital records offices with the goals of disseminating recommendations to eliminate preventable maternal mortality and disparities [28]. As of 2023, 49 states, the District of Columbia, New York City, Philadelphia, and Puerto Rico had formal MMRCs or a legal requirement to review pregnancy-related deaths [29].

Deep dives into individual cases by review committees situate diagnostic errors within the complex provider and system errors that contribute to pregnancy-related harm. For example, the New York State Department of Public Health published a summary of the 117 pregnancy-related deaths (within one year of delivery) in 2018. Of these, 78% were deemed preventable. By category of obstetric cause of death, 100% of deaths due to hemorrhage, cardiomyopathy, and mental health were determined to be preventable. In examining the factors contributing to obstetric deaths in New York State, provider-level aspects including medical knowledge, clinical assessment, skill, quality of care, care coordination and continuity, and delay in care were contributory in 36.8% of cases. In 21.9% of cases, facility-level issues played a role, including clinical skill, quality of care, care coordination and continuity, policies and procedures, and equipment and technology. In 19.4% of cases, system-level factors were at play, such as knowledge, clinical skill, quality of care, and structural racism [6].

Case reviews, however, rely on deaths or adverse outcomes to be referred for an institutional incident review or, in the case of a local or state MMRC, identified from vital records. Thus, a well-functioning referral infrastructure must be in place. At the institutional level, case identification requires patient quality and safety teams to have adequate resources and processes in place. While institution-level adverse event reporting and reviews capture pregnancy-related morbidity, the majority of maternal mortality review committees focus only on deaths and thus do not capture non-fatal diagnostic errors.

(5) Malpractice and administrative claims databases

A fifth opportunity to identify diagnostic error rates and harms is the use of malpractice claims databases [10,30]. For example, a study by Gupta et al. queried the US National Practitioner Database for malpractice claims and utilized multivariable logistic regression to identify patient and provider factors associated with inpatient diagnosis-related paid claims [31]. Approximately 22% of all claims were diagnosis-related, associated with USD 5.7 billion in payments over the study period. They also reported patient and provider characteristics associated with diagnosis-related claims, such as patient age and physician level of training.

To our knowledge, there have not been similar studies in obstetrics. However, given examples of these studies in other fields and the existence of malpractice databases that include obstetric cases, this research method would be a feasible undertaking. The major disadvantage of this method is the lack of detail regarding the clinical case and surrounding health systems processes that contributed to the diagnostic error and harm. However, the use of malpractice databases to identify diagnostic errors in obstetrics presents an opportunity for the high-level understanding of diagnostic error rates by obstetric conditions, patient and provider factors associated with diagnostic errors, and estimates of the financial impact of these harms.

In any of these research methods, ascertaining diagnostic error rates and associated harms can be challenging. Diagnostic errors are tricky: they are rarely recognized in real time. Instead, the majority surface in retrospective review by other clinicians, or adverse event reporting. In this sense, they can remain elusive. Measuring and studying diagnostic errors in obstetrics can also be challenging due to the unique and varied landscape of care. Obstetrics comprises the ambulatory, emergency, and inpatient settings, with labor and delivery representing a distinctive type, but not the only type, of inpatient care. Each of these environments may contribute to a different way to monitor and study diagnostic errors. The transitions between care environments present their own opportunities for diagnostic errors [32]. Moreover, pregnancy represents a time-limited episode of care, and many patients transition after pregnancy to other providers and care models, back to their primary care or specialty care, general emergency department use, or no care at all. As pregnancy-related harm attempts to capture morbidity and mortality within one year of pregnancy, the study of diagnostic errors in obstetrics must include the care transitions beyond the fourth trimester, or the critical period of the twelve weeks following birth.

In addition, diagnostic errors, particularly in obstetrics, are not well defined. Without standardized definitions, quality improvement initiatives and research studies are difficult to aggregate and compare. It is helpful to return to the National Academy of Sciences definition of diagnostic errors as the failure to establish an accurate and timely explanation of the patient’s health problem or to communicate that explanation to the patient [6]. This is a thorough but complex definition that captures the accuracy of diagnosis, the time to diagnosis, and the communication of the diagnosis to the patient. Metrics for obstetric diagnostic errors should consider all three of these facets.

## 3. Discussion: Promoting Diagnostic Excellence in Obstetrics

**Figure 1:**
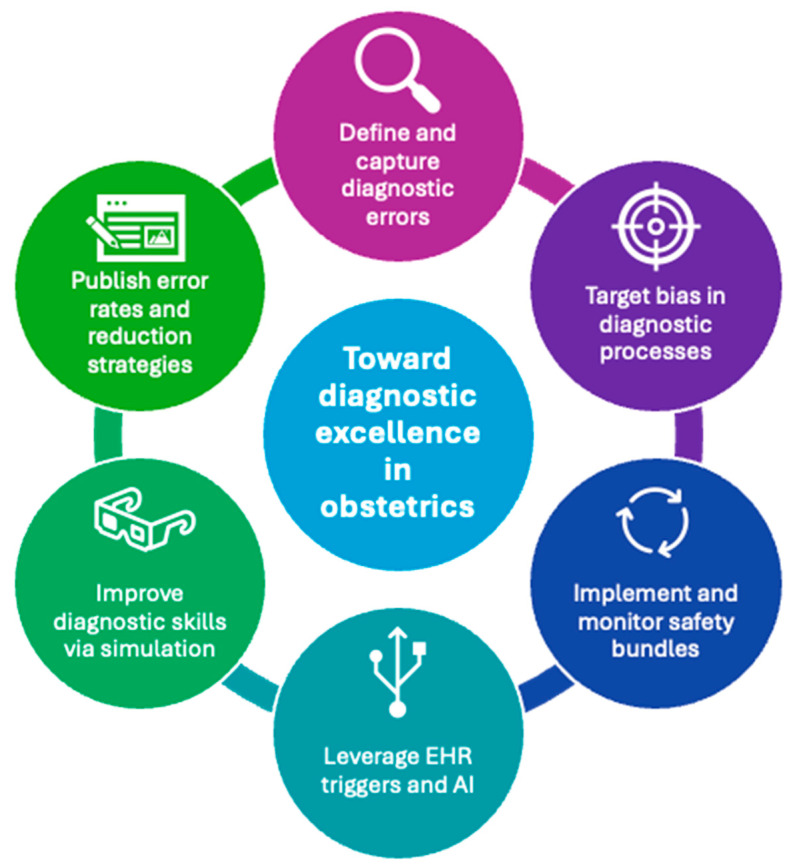
Steps toward diagnostic excellence in obstetrics. EHR = electronic health record, AI = artificial/augmented intelligence.

To reduce diagnostic errors and strive for diagnostic excellence in obstetrics, we must answer two questions: How often are we getting the obstetric diagnosis right? And, when we get it wrong, why? To this end, we propose a framework for identifying and reducing diagnostic errors for future study. We suggest several goals to pursue diagnostic excellence in the obstetric community (Figure 1).

First, we must define and capture diagnostic errors. Metrics for diagnostic errors should target both mortality and morbidity. One category of metrics includes comparing diagnoses, for example, autopsy diagnoses compared to death certificate diagnoses, inpatient obstetric admission versus discharge diagnoses, inpatient readmission diagnoses compared to discharge diagnoses, and admission diagnoses within close proximity to an outpatient visit compared to outpatient visit diagnoses. Assessment of different diagnoses within a short timeframe can flag potential missed or delayed diagnoses. In fact, discrepancies between admission and discharge diagnoses have been shown to be associated with a longer length of stay, greater odds of intensive care unit stay, readmission, and mortality [33,34,35]. A second category of diagnostic errors focuses on the communication of diagnoses to patients and patients’ understanding of their diagnoses. This category of metrics relies upon patient feedback regarding their care and care team communication. Survey reports in other fields include inquiries into metrics such as time from symptom onset to diagnosis, alternate diagnoses received, poor or inadequate communication of diagnoses [36,37,38]. While some patient metrics may be captured in existing patient experience surveys, the creation of standardized patient and family feedback forms, particularly for significant cases of morbidity and mortality flagged via incident review, may assist in understanding this important component of diagnostic errors.

Second, bias in diagnostic errors must be targeted. Medical errors can result when clinical decision making is affected by cognitive biases or implicit systematic errors in thinking. The use of race in clinical algorithms and treatment guidelines can result in diagnostic errors. Personal provider bias can also play a conscious or subconscious role in misdiagnosis or can delay diagnosis [39]. In particular, racial bias among medical providers can contribute to worse health outcomes [40,41,42]. Bias identification and prevention training should be standard in all obstetric practices. Specific to the case review of errors, a “cognitive autopsy”, or intentionally reviewing whether bias played a role in the error, should be formalized in all retrospective case reviews for both institutional quality and safety committees and state maternal mortality review boards [39].

Third, a diagnostic error lens should be built into health system quality and safety frameworks. As evidence shows that simulations can identify medical errors, obstetric simulation training should incorporate arriving at a diagnosis and include clear feedback when cases are misdiagnosed [43]. Implementation of obstetric safety bundles such as those by the Alliance for Innovation on Maternal Health and the Safe Motherhood Initiative provide structured processes for management of obstetric care, but are often crafted around a diagnosis, meaning they are clinically applied after arriving at a diagnosis for a patient, for example, severe hypertension or postpartum hemorrhage. Adding diagnostic care pathways to safety bundles to flag patients early in a workup that should ultimately fall into a safety bundle process represents an important step of successful safety bundle implementation. The Safe Motherhood Initiative Maternal Early Warning System’s overarching algorithm triggered by abnormal vital signs combined with an individual patient’s clinical presentation, risk factors, and additional diagnostic tests provides a likely diagnosis by safety bundle (sepsis, hemorrhage, hypertension, or venous thromboembolism) and represents a step toward diagnostic pathways for obstetric emergencies [44]. Special attention should be paid to the frequent transitions of care in obstetrics, as highlighted in the Alliance for Innovation on Maternal Health’s Postpartum Discharge Transition Bundle. This bundle outlines readiness, recognition and prevention; response, reporting and systems learning; and respectful, equitable and supportive care for the critical immediate postpartum period from hospital discharge to outpatient obstetrical care [45]. Diagnostic errors during postpartum visits and readmissions can cause significant harm and deserve particular attention.

Fourth, health technology should be leveraged to flag and discover missed diagnostic errors. Electronic health records can be used to help screen for diagnostic errors via the implementation of electronic triggers or pre-programmed tools to recognize signals of a likely error or adverse event [46]. Electronic triggers have been used successfully to identify other errors, such as wrong-patient orders and medication errors [47]. Triggers can also be applied to screen for diagnostic errors. In obstetrics, potential electronic triggers of diagnostic errors could include a drop in hematocrit not already associated with a diagnosis of postpartum hemorrhage, such as in the study example above. As with event reporting systems, to best strive for diagnostic excellence, triggers flagging potential diagnostic errors should be built into electronic health record and patient monitoring workflows and dashboards from the outset.

The promise of health technology to improve obstetric diagnosis lies in the potential for prospective healthcare team alerts. In obstetrics, electronic triggers for early warning signs such as for hypertension have shown a significant benefit in promoting early evaluation to avoid significant adverse outcomes. Artificial intelligence (AI) continues to disrupt the healthcare landscape, and utilizing AI in the diagnostic errors space provides promising ways to predict errors and reduce harm. By bringing together information for providers from electronic fetal monitoring signals to vital signs to laboratory results, AI could greatly improve the diagnostic accuracy in real time [48].

Lastly, there is a dearth of literature on diagnostic errors in obstetrics. Sharing innovative approaches to detect, study, and prevent obstetric errors via publication in journals will be essential to moving the needle in diagnostic excellence. Maternal mortality review committees should also be encouraged to publish their findings not only in reports but in the literature, and convenings of these committees at national meetings should directly address strategies for the identification of diagnostic errors. Journals should recognize the importance of publishing studies that seek to promote diagnostic excellence in obstetrics.

## 4. Conclusions

In conclusion, despite valiant efforts across the obstetric community, we remain in an obstetric morbidity and mortality crisis. Behind the rates and cases of adverse obstetric outcomes are birthing people, newborns, and their families and support networks. We must continue to learn from their experiences. As we continue to iterate on processes to review cases of morbidity and mortality, provider-level factors have emerged as a significant contributor and thus an opportunity for improvement. Within provider-level factors and intersecting closely with systems issues lie obstetric diagnostic errors, under-recognized and under-researched. Specifically, cases of pregnancy-related deaths should be referred to medical examiners for autopsy, and the pathologic cause of death compared to the clinical and existing institutional case reviews and maternal mortality case reviews should incorporate the identification of diagnostic errors and any contributory bias and publish their findings and recommendations. Institutions should include questions regarding diagnostic errors in patient surveys and leverage quality and safety teams to focus on diagnostic errors. Areas of focus may include implementing and evaluating obstetric safety bundles, utilizing automated queries and triggers to detect diagnosis discrepancies between transitions of care and readmissions, and clinical criteria for common obstetric morbidities—including hypertensive diseases of pregnancy and postpartum hemorrhage. Institutional simulation and education teams may consider running scenarios to ascertain and target common diagnostic errors. A focus on diagnostic errors requires a foundation of psychological safety, engaged leadership, and adequate resources that, though beyond the scope of this paper, is essential for success [48]. If properly supported, the research methods and error reduction strategies outlined above have the potential to move our field toward diagnostic excellence and improved patient care and outcomes. A future evaluation of these methods and their impact on patient outcomes is necessary to determine the most effective strategies to reduce diagnostic error and harm in obstetrics.

## Figures and Tables

**Table 1:** Pros and cons of selected research methods of diagnostic errors in obstetrics.

Research Method	Pros	Cons
**Clinicopathologic autopsy studies**	+ Provide objective pathologic cause of death to compare with clinical diagnosis	- Capture only mortality, not morbidity - Rely on referrals to and acceptance from medical examiners
**Retrospective chart review based on clinical criteria**	+ Allow for deep dive into clinical and systems-based issues of misdiagnosis + Can produce automated electronic triggers and frameworks	- Rely on searchable clinical criteria (ex: vitals, laboratories) that may not be clear for complex diagnoses
**Obstetric simulation and standardized patients**	+ Identify diagnoses with high error rates their root causes + Enable real-time feedback for clinicians and broader multidisciplinary teams	- Do not provide real-world data on cases or diagnostic error rates
**Pregnancy-related case reviews (incident reporting and maternal mortality reviews)**	+ Yield thorough and individualized case reviews + Can situate diagnostic errors within complex systems	- Rely on reporting systems to identify cases for review - State boards typically review only deaths
**Malpractice and administrative claims database queries**	+ Represent large datasets from a variety of hospitals + Allow for estimating financial impact of diagnostic harm	- Include few clinical case details - Lack information on systems-level issues associated with missed diagnoses
